# Subcutaneous versus Intravenous Administration of Rituximab: Pharmacokinetics, CD20 Target Coverage and B-Cell Depletion in Cynomolgus Monkeys

**DOI:** 10.1371/journal.pone.0080533

**Published:** 2013-11-12

**Authors:** Cheng-Ping Mao, Martin R. Brovarney, Karim Dabbagh, Herbert F. Birnböck, Wolfgang F. Richter, Christopher J. Del Nagro

**Affiliations:** 1 Discovery Inflammation, Roche Palo Alto, Palo Alto, California, United States of America; 2 Drug Metabolism and Pharmacokinetics, Roche Basel, Basel, Switzerland; 3 Discovery Oncology, Genentech Incorporated, South San Francisco, California, United States of America; University of North Carolina at Chapel Hill, United States of America

## Abstract

The CD20-specific monoclonal antibody rituximab (MabThera^®^, Rituxan^®^) is widely used as the backbone of treatment for patients with hematologic disorders. Intravenous administration of rituximab is associated with infusion times of 4–6 hours, and can be associated with infusion-related reactions. Subcutaneous administration of rituximab may reduce this and facilitate administration without infusion-related reactions. We sought to determine the feasibility of achieving equivalent efficacy (measured by endogenous B-cell depletion) and long-term durability of CD20 target coverage for subcutaneously administered rituximab compared with intravenous dosing. In these preclinical studies, male cynomolgus monkeys were treated with either intravenous rituximab or novel subcutaneous formulation of rituximab containing human recombinant DNA-derived hyaluronidase enzyme. Peripheral blood samples were analyzed for serum rituximab concentrations, peripheral B-cell depletion, and CD20 target coverage, including subset analysis according to CD21+ status. Distal lymph node B-cell depletion and CD20 target coverage were also measured. Initial peak serum concentrations of rituximab were significantly higher following intravenous administration than subcutaneous. However, the mean serum rituximab trough concentrations were comparable at 2 and 7 days post-first dose and 9 and 14 days post-second dose. Efficacy of B-cell depletion in both peripheral blood and distal lymph nodes was comparable for both methods. In lymph nodes, 9 days after the second dose with subcutaneous and intravenous rituximab, B-cell levels were decreased by 57% and 42% respectively. Similarly, levels of peripheral blood B cells were depleted by >94% for both subcutaneous and intravenous dosing at all time points. Long-term recovery of free unbound surface CD20 levels was similar, and the duration of B-cell depletion was equally sustained over 2 months for both methods. These results demonstrate that, despite initial peak serum drug level differences, subcutaneous rituximab has similar durability, pharmacodynamics, and efficacy compared with intravenous rituximab.

## Introduction

The CD20-specific monoclonal antibody (mAb) rituximab (MabThera^®^, Rituxan^®^) was the first mAb approved for use in the treatment of cancer. Rituximab is widely used as the backbone of treatment for patients with non-Hodgkin’s lymphoma (NHL) and chronic lymphocytic leukemia (CLL) [1,2]. Rituximab is also approved in combination with methotrexate in adult patients with moderately to severely active rheumatoid arthritis who have inadequate response to one or more tumor necrosis factor antagonist therapies, and in combination with glucocorticoids for adult patients with Wegener’s granulomatosis and microscopic polyangiitis [3]. 

In hematologic malignancies, rituximab is currently administered by intravenous (IV) infusion at a dose of 375 mg/m^2^ (NHL) or 500 mg/m^2^ (CLL) body surface area [3]. The initial rate for first infusions of rituximab is 50 mg/h and, in the absence of infusion-related reactions, this can then be increased by 50 mg/h increments every 30 minutes, to a maximum of 400 mg/h. Subsequent doses of rituximab can be infused at an initial rate of 100 mg/h, and increased by 100 mg/h increments at 30-minute intervals, to a maximum of 400 mg/h [3]. As a result, typical total infusion times average 6 hours for the first infusion and 4 hours for subsequent infusions.

IV infusions require inconvenient clinic visits for patients and increased demand on healthcare providers, along with increased safety risks and healthcare-related costs [4-7]. Although most infusion-related reactions are mild to moderate and occur predominantly with the first infusion [1,3], they lead to even longer and more frequent clinic and hospital visits, and an increased burden on healthcare resources [4-7]. The disadvantages of IV infusion are most keenly felt by patients who require numerous and regular rituximab infusions; follicular lymphoma patients, for example, receive rituximab maintenance treatment every 2 months (starting 2 months after the last dose of induction therapy) for a maximum of 2 years [3]. 

The subcutaneous (SC) administration of mAbs such as alemtuzumab and adalimumab has demonstrated several benefits over traditional IV infusions, including notable reductions in administration time, infusion-related reactions and related healthcare costs, and increased patient convenience [8-10]. Alemtuzumab SC is given as a 30 mg dose, split into two injections, each of 1.5 ml [9]; however, the dose required for rituximab is much higher, necessitating larger dose volumes that can be a limitation to SC administration [3,11]. Currently, a novel SC formulation of rituximab containing human recombinant DNA-derived hyaluronidase enzyme (rHuPH20) is under investigation to overcome the dose volume limitation. rHuPH20 functions as a permeation-enhancing agent that temporarily depolymerizes the local interstitial matrix component hyaluronan at the SC injection site and thereby increases the dispersion, absorption, and bioavailability of larger volumes of co-injected drugs [12,13].

It is important for rituximab SC administration to result in effective dissemination from the injection site in order to achieve adequate serum concentrations for rituximab distribution to lymphoid tissues throughout the body, as this is critical for the efficacy of rituximab in targeting malignant B cells for depletion [14]. In these preclinical studies we evaluated the pharmacokinetics (PK) of a single dose (20 mg/kg) of rituximab SC and compared the PK, pharmacodynamics (PD) of CD20 target coverage, and B-cell depletion efficacy associated with two 10 mg/kg doses, given 7 days apart, for rituximab IV versus rituximab SC in cynomolgus monkeys, a widely used model to study therapies aimed at B-cell depletion [15,16]. The latter dosing schedule is approximately equivalent to the standard rituximab dose in humans [15]. Two different circulating subsets of cynomolgus monkey B cells have been previously identified: CD20high/CD40low/CD21- (CD21- B cells) and CD20low/CD40high/CD21+ (CD21+ B cells). These differ in their *in vivo* and *in vitro* susceptibility to rituximab; the CD21- B-cell subset is more easily depleted than the CD21+ B-cell subset that is phenotypically closer and responds in an equivalent way to human peripheral blood B cells exposed to rituximab [15,16]. Peripheral blood mononuclear cell (PBMC) CD21- and CD21+ B-cell subset depletion in response to rituximab SC or IV administration was examined. 

## Methods

### Drug administration and tissue collection

In an initial PK study, three male cynomolgus monkeys (*Macaca fascicularis*) older than 5 years of age (6 to 8 kg) were treated with rituximab (20 mg/kg) formulated by inversion in PBS containing rHuPH20 at 120 mg/ml (6000U/ml) given in the interscapular region (F. Hoffmann-La Roche). In the subsequent comparative pharmacologic study, eleven male cynomolgus monkeys (approximately 4 years of age and 3 to 5 kg) were treated twice (on days 0 and 7) with either a rituximab SC formulation containing rHuPH20 or the traditional IV formulation (n = 4 per each drug administration groups; both 10 mg/kg × 2, and n = 3 in the PBS vehicle control group; F. Hoffmann-La Roche); the site of the SC injection of rHuPH20 preformulated rituximab was the thigh. 

Axillary lymph node biopsies (i.e., from a lymph node outside of the lymphatic drainage pathway from the SC injection site) were collected 7 days prior to the first dose (baseline) and 9 days after the second dose. Lymph nodes were mechanically dissociated into ice-cold Roswell Park Memorial Institute 1640 growth medium containing 10% fetal bovine serum, then resuspended in fluorescence-activated cell sorting (FACS) staining buffer containing phosphate-buffered saline (PBS) with 1% bovine serum albumin (BSA) and 0.05% sodium azide. In the initial PK study, peripheral blood was taken periodically over 84 days by venous blood draw. In the comparative pharmacologic study, peripheral blood was taken 7 days prior to the first dose (baseline), 1 hour, 2 days, and 7 days after the first dose, and 1 hour and 2, 9, and 14 days after the second dose. Additional blood samples were collected by venous blood draw and analyzed weekly for 2 months (63 days) following the final dose. For isolation of PBMCs, peripheral blood was diluted 2× in PBS with BSA and Ficoll purified. The cells were resuspended in FACS staining buffer prior to fluorescence staining. Flow collection was performed on a BD™ LSR II flow cytometer (BD Biosciences, Franklin Lakes, NJ, USA) and analysis was performed using FlowJo software (Tree Star Inc., Ashland, OR, USA). 

### Ethics Statement

These studies were reviewed and approved by The Roche Group ethical committee and were carried out in strict accordance with the Swiss Animal Protection Act, the EU Directive on the Protection of Animals used for Scientific Purposes (2010/63/EU), and the European Convention for the protection of vertebrate animals used for experimental and other scientific purposes (ETS 123) by the Council of Europe with under approved protocol #08-5244, and under the Animal Welfare Act and Animal Welfare Regulations of the US Department of Agriculture and the Institute for Laboratory Animal Research (ILAR) guide for the care and use of laboratory animals with IACUC approved protocol #2006-76. All efforts were made to maintain an enriched living environment and minimize procedural suffering; compound administration, lymph node biopsies and serological collection were all performed under sedation with anesthesia. Animals were individually housed in stainless steel, one over one caging. 12 hour light/dark cycles, temperature and humidity including 10-15 air changes per hour were continuously monitored utilizing an environmental Watchdog® System (Edstrom Industries). Reverse osmosis filtered water was accessible ad libitum, while animals were fed certified chow biscuits twice daily and were provided both tactile and food enrichment in the form of various vegetables and fruits, foraging mix boards, as well as in and on cage tactile enrichment devices daily. Additional environmental enrichment in the form both natural auditory background sounds and visual nature video enrichment were provided daily as well as scheduled access to open enrichment rooms and 360 degree bay-window viewing and jungle patterned cages as per veterinary evaluation of ongoing animal disposition. As the work was non-terminal and minimally invasive with only serological sampling and peripheral lymph node biopsies taken, no animals were euthanized in the conduct of these studies.

### Pharmacokinetic analysis

Serum rituximab levels were analyzed by a validated enzyme-linked immunosorbent assay. Standard curves were created using stock rituximab diluted in cynomolgus monkey serum. Diluted standards and diluted serum samples (10 µl serum; ≥1:100 dilution in assay diluent) were transferred to high protein affinity 96-well plates coated with polyclonal goat anti-rituximab antibodies (Genentech, Inc., South San Francisco, CA, USA). The plates were washed, and bound rituximab was detected by incubation with goat anti-mouse IgG F(ab’)2 conjugated to peroxidase (Jackson ImmunoResearch, Inc., West Grove, PA, USA; 115-036-072). Bound peroxidase was detected through a chromogenic reaction, with absorbance (450–630 nm) proportional to the amount of rituximab present in the sample. Data acquisition and processing were performed using the software packages SoftMax^®^ Pro version 3.1.2 (Molecular Devices Corporation, Sunnyvale, CA, USA) and Microsoft^®^ Office Excel 2003 (Microsoft Corporation, Washington, DC, USA). The lower limit of quantification for the assay was 5.0 ng/ml (serum concentration: 500 ng/ml) with a calibration range of 2.5–160 ng/ml. Precision of the assay, determined as the coefficient of variability on quality control sample analysis, was ≤7.3%. The accuracy of the assay was between 87.0% and 89.4%. Statistical analysis for calculation of p values was determined by an two-tailed unpaired student t test (homoscedastic – assuming equal variances). Maximum concentration, time point at maximum concentration, and area under the curve from time zero to infinity (AUC[0-∝]) were estimated by noncompartmental analysis, using the PK evaluation program ToxKin^TM^ 3.5.3 (Entimo AG, Berlin, Germany).

### Flow cytometry

Multicolor FACS analysis was performed for CD20-independent B-cell identification and CD21+ and CD21- B-cell subset identification and to measure the depletion of B cells in both PBMC and distal lymphoid tissue, and target coverage of B-cell surface CD20. mAbs used for the staining of cells were anti-CD21 plus fluorescein isothiocyanate (clone LT21; BioLegend, San Diego, CA, USA), anti-CD40 plus phycoerythrin (PE; clone HB14; Caltag, Burlingame, CA, USA), anti-CD4 plus allophycocyanin (APC; clone S3.5; Caltag), anti-CD16 plus PE-cyanine 5 (PE-Cy5; clone 3G8; BD Pharmingen, San Diego, CA, USA), anti-CD20 plus PE-Cy7 (clone L27; BD Pharmingen), and anti-CD3 plus APC-Cy7 (clone SP34-2; BD Pharmingen).

#### CD20-independent B-cell identification

Identification of B cells by flow cytometry in cynomolgus monkeys usually relies on the classical method of B-cell gating using anti-CD20 antibodies. However, with rituximab administration, CD20 is fully or partially covered by unlabeled rituximab, thus obscuring CD20-dependent B-cell identification and requiring a CD20-independent means of identifying B cells for quantification. Since antibodies against CD19, the other B-cell-specific marker commonly used to identify human B cells, are not cross-reactive between the human and cynomolgus monkey, a novel CD20-independent gating paradigm to efficiently identify cynomolgus monkey B cells was used. To identify B cells independent of CD20, cells were lymphocyte-gated, which accounts for approximately 25% of B cells, and then gated on CD4-/CD3- non-T cells, resulting in an approximately 70% pure B-cell population. CD4-/CD3- cells were then sub-gated on CD40+/CD16- to achieve an approximately 98% pure B-cell population when verified using fluorescently labeled anti-CD20 antibodies on untreated PBMC cells (see results section). Using this four-color stain it was possible to use the CD4+/CD3+ T cells for ratiometric quantitative analysis between samples. 

#### B-cell depletion and CD20 target coverage

The efficacy of B-cell depletion was determined in lymph nodes and PBMCs by the ratio of B cells to CD4+ T cells (CD4+/CD3+). B cells (CD3-/CD4-/CD16-/CD40+) were identified by flow cytometry, as described above, and verified by CD20 and CD21 expression on equivalent phosphate buffered saline (PBS) vehicle-treated samples. Averages were calculated as mean value for all samples ± standard deviations (SD) calculated using the appropriate n values for tested groups. 

Rituximab target coverage was determined by *ex vivo* flow staining for unbound surface CD20 on B cells identified as described above, using flow cytometry, with a fluorescently labeled anti-CD20 antibody that binds to the same epitope as rituximab. Coverage was quantified as mean fluorescence intensity and compared with PBS vehicle samples treated with (full coverage) or without (no coverage) 10 µg/ml rituximab *ex vivo* for 30 minutes at 4°C prior to staining with a fluorescently labeled anti-CD20 antibody. Treating purified PBMCs and lymph node cells *ex vivo* with rituximab (10 µg/ml) prior to staining was an effective way to fully cover the available CD20, resulting in mean fluorescence staining levels nearly equivalent to those of non-B cells (CD4+ T cells) (see Results section). Averages were calculated as mean value for all samples ± standard deviations (SD) calculated using the appropriate n values for tested groups. 

#### CD21+ and CD21- B-cell subset identification

A fluorescently labeled anti-CD21 antibody was used to distinguish the two different circulating subsets of cynomolgus monkey B cells (CD21- B cells and CD21+ B cells) within the CD3-/CD4-/CD16-/CD40+ gated B cells (see Results section). 

## Results

### Pharmacokinetic analysis

**Figure 1:**
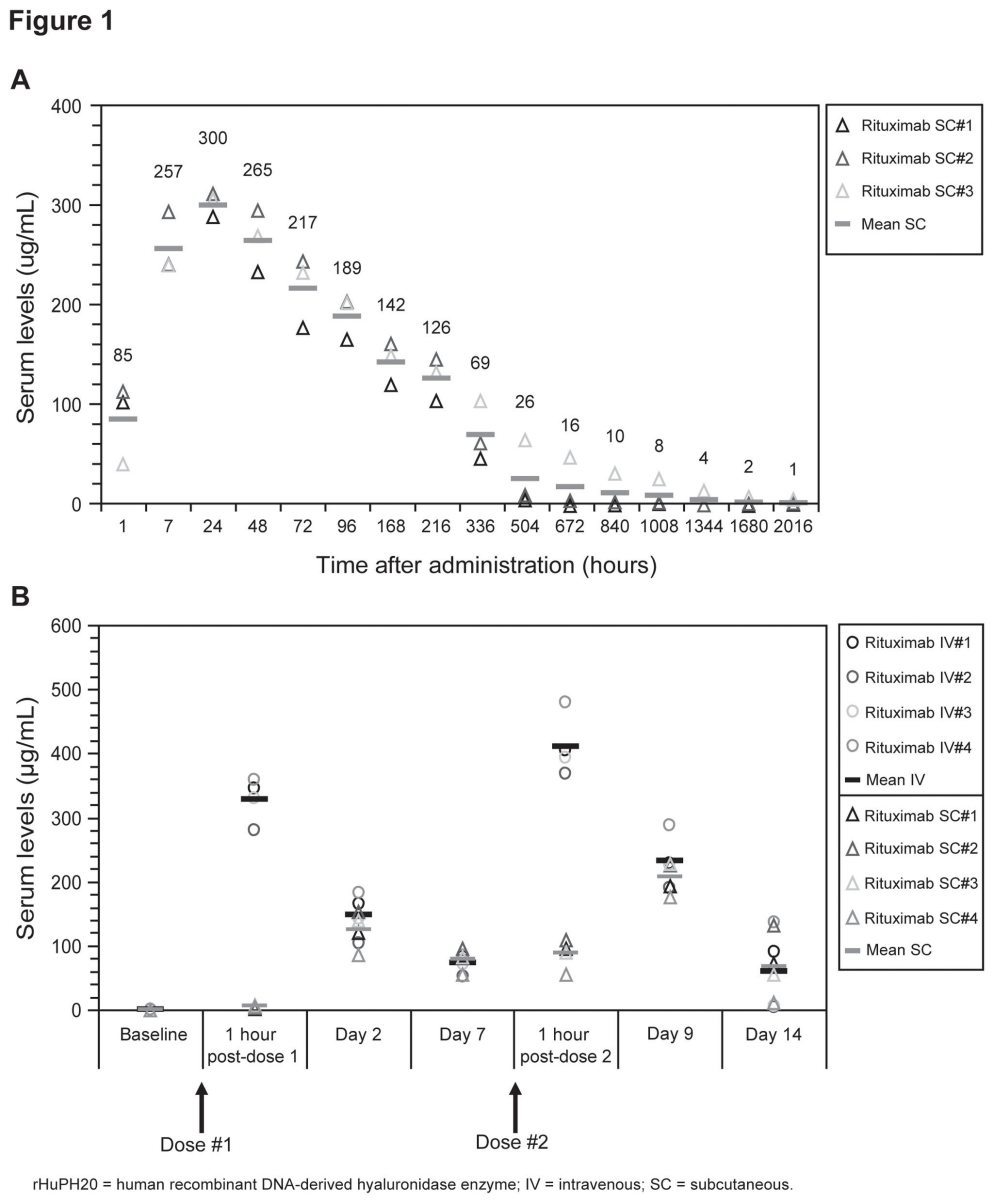
Pharmacokinetics of serum rituximab in cynomolgus monkeys. PK analysis of serum rituximab levels after (**A**) a single SC dose of rituximab preformulated in rHuPH20 administered at 20 mg/kg (Individual animal samples with mean score shown (n=3)), and (**B**) 2 × 10 mg/kg doses of SC (preformulated in rHuPH20) or standard IV rituximab, given 7 days apart (Individuals animal samples with mean shown (n=4)).

In the initial pharmacokinetic analysis in cynomolgus monkeys, a single rituximab SC dose (20 mg/kg) showed elevated drug levels (± SD) within 1 hour of administration (85 ± 40.15 µg/ml), reaching peak serum drug level within 24 hours (300 ± 10.82 µg/ml) and gradually decreasing in concentration over time as measured up to 84 days (2016 hours; Figure 1A). AUC[0-∝] (± SD) was 72,600 ± 27,400 µg·h/ml. In a subsequent pharmacologic study cynomolgus monkeys were treated twice (on days 0 and 7) with either rituximab IV or SC (both routes at 10 mg/kg/dose). Consistent with the mode of delivery, in the comparative analysis mean serum rituximab concentrations (± SD) were significantly higher 1 hour after each dose in the rituximab IV group compared with the rituximab SC group (1 hour post-dose 1: 328.5 ± 34.4 µg/ml IV versus 5.2 ± 2.4 µg/ml SC, P = 0.000001; 1 hour post-dose 2: 411.0 ± 47.8 µg/ml IV versus 87.6 ± 23.2 µg/ml SC, P = 0.00002) (Figure 1B; Table 1). However, mean serum rituximab trough concentrations were comparable 2 and 7 days after the first dose and 9 and 14 days after the second dose of rituximab SC or IV, despite the different mode and formulation of administration (Figure 1B; Table 1) (non-significant p values). Mean trough levels continued to be comparable up to 69 days after the second dose (data not shown). The SC dose of 20 mg/kg in the initial study reached a maximal concentration (C_max_) of 300 μg/ml at day 2, comparable to the C_max_ achieved at 1 hour post a single 10 mg/kg IV dose in the second study (328.5 ± 34.4 µg/ml). 

**Table 1:** PK analysis of serum rituximab levels after 2 × 10 mg/kg doses of SC (preformulated within rHuPH20) or standard IV rituximab.

		**Serum rituximab concentration (μg/ml) mean ± standard deviation**		
**Dose**	**Time point**	**Rituximab SC (n = 4)**	**Rituximab IV (n = 4)**	**P value (Student *t* test)**
Post-dose 1	1 h	5.2 ± 2.4	328.5 ± 34.4	P= 0.000001
	Day 2	124.7 ± 28.7	148.0 ± 33.6	NS (>0.3)
	Day 7	79.0 ± 16.3	72.3 ± 14.6	NS (>0.5)
Post-dose 2	1 h	87.6 ± 23.2	411.0 ± 47.8	P = 0.00002
	Day 9	206.3 ± 25.4	232.8 ± 40.2	NS (>0.3)
	Day 14	68.0 ± 50.3	59.5 ± 64.9	NS (>0.8)

Statistically significant P value <0.001; **NS** = not significant; **IV** = intravenous; **SC** = subcutaneous; **rHuPH20** = human recombinant DNA-derived hyaluronidase enzyme

### Flow cytometry analysis

**Figure 2:**
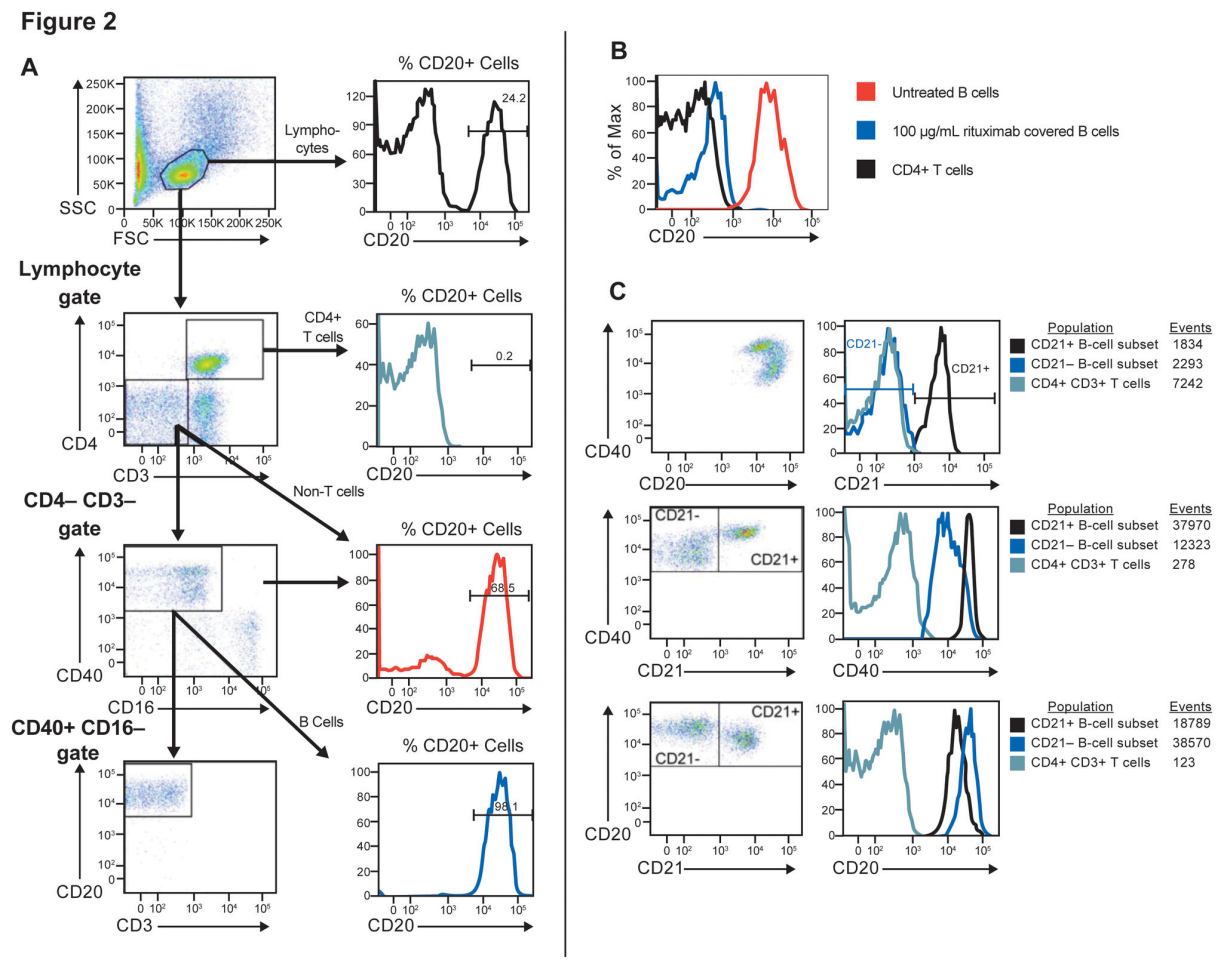
CD20 target coverage in B-cells and according to CD21+ status. Paradigms for flow cytometry staining: (**A**) CD20-independent identification of B cells using fsc/scc lymphocyte gating followed by CD4/CD3 negative gating and CD40 positive CD16 negative gating shows a progressively increasing specific B cell population; (**B**) Free surface CD20 levels on B cells (as identified in A) with and without rituximab treatment to show target coverage of CD20 compared to CD4+/CD3+ T cells; (**C**) Identification of CD21+ and CD21- peripheral blood B-cell subsets (within the CD4-/CD3-/CD16-/CD40+ gated B cells as identified in A) showing CD21, CD40 and CD20 levels of the subsets.

To facilitate the evaluation of B-cell depletion and determine target coverage after administration of a drug that masks surface CD20, we developed a method to identify B cells in cynomolgus monkeys without using anti-CD20 as the definitive B-cell marker. We found anti-human and anti-mouse CD19 antibodies were weakly cross-reactive with cynomolgus CD19; at the time we conducted this study, anti-cynomolgus CD19 antibodies were not commercially available, so we utilized an exclusion gating coupled with a CD40-positive gating paradigm (Figure 2A) for flow cytometric B-cell identification. Gating on CD3-/CD4- cells converts an originally 24.2% B-cell population into a 68.5% pure population and then subsequent gating on CD40+/CD16- cells results in CD20-independent identification of a highly pure B-cell population of 98.1%. We utilized the CD3+/CD4+ T-cell population as a reference population for ratiometric B-cell enumeration. Utilizing this gating paradigm, we could then stain with a competitive binding, fluorescently labeled anti-CD20 antibody to measure free surface CD20 levels and determine CD20 target coverage by loss of mean fluorescence intensity (Figure 2B). We were able to identify and enumerate CD21+ and CD21- peripheral blood B-cell subsets by staining with an additional anti-CD21 antibody (Figure 2C).

### Lymph node B-cell analysis

**Figure 3:**
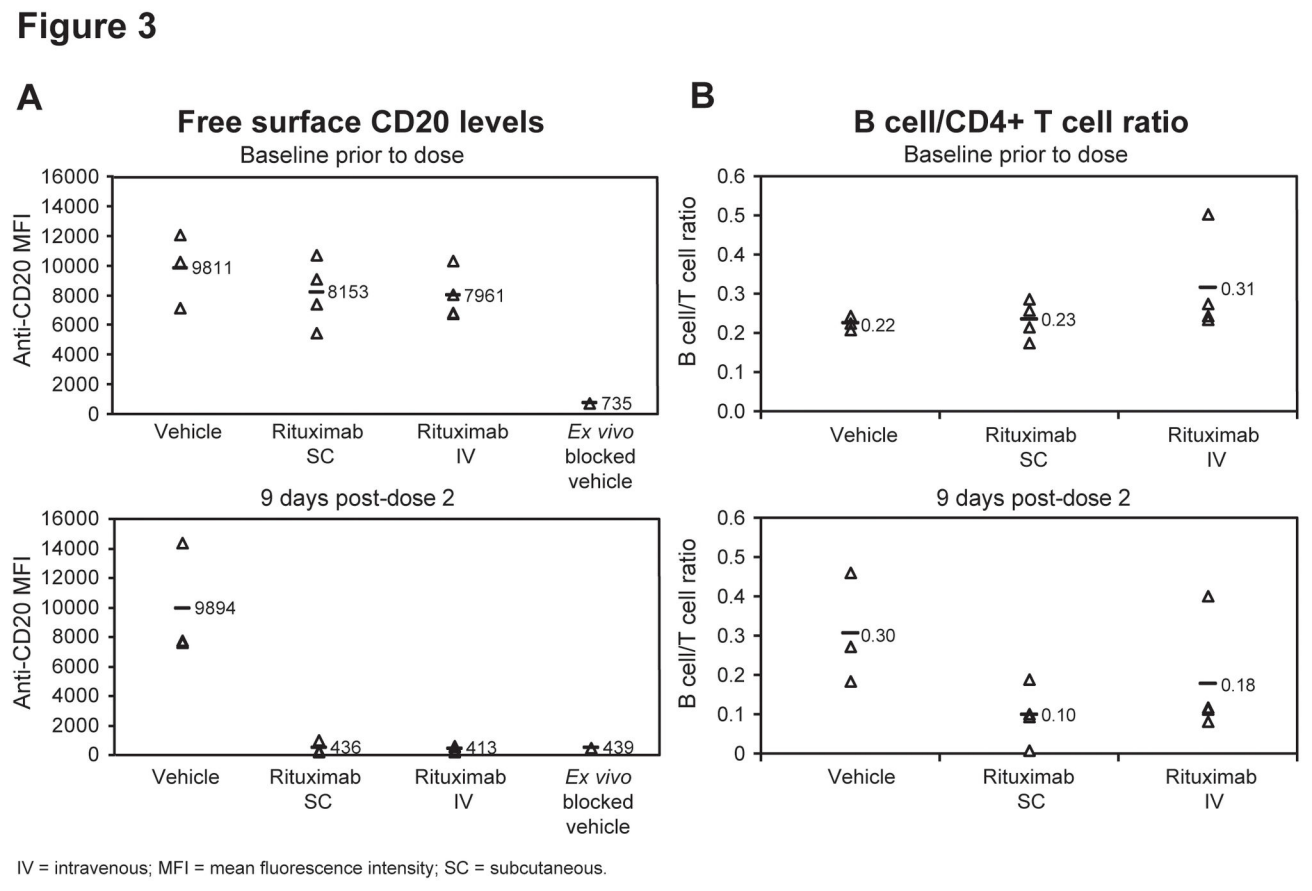
CD20 target coverage and B-cell depletion in lymph nodes. Lymph node analysis of (**A**) CD20 target coverage as determined by flow cytometric staining for free surface CD20 levels on (B cells identified as in Figure 2A) and (**B**) depletion of those lymph node B-cellsat baseline and 9 days after second dose of subcutaneous or intravenous rituximab as determined by a ratio to CD4+/CD3+ T cells (Individuals animal samples with mean shown (n=4 rituximab treated groups, n=3 PBS vehicle treated groups)).

Rituximab SC and IV both fully covered distal lymph node B-cell surface CD20, reducing the staining of free surface CD20 by 95% compared to baseline measurements, reaching levels equivalent to those achieved with a saturating *ex vivo* block (Figure 3A). Both administration modes also depleted lymph node B cells to a similar extent 9 days after the second dose (SC: 57% versus IV: 42%) (Figure 3B). We could not perform CD21+ versus CD21- B-cell subset analysis, as the CD21- B-cell subset was not present in lymph node tissues of tested animals (data not shown). We believe that the CD21- B-cell subset represents early circulating immature B cells recently egressing from the bone marrow. This is similar to the situation in mice, where immature CD21- B cells mature into CD21+ mature B cells prior to gaining the ability to migrate into lymph nodes and splenic lymphoid follicles [17]. 

### PBMC B-cell analysis

**Figure 4:**
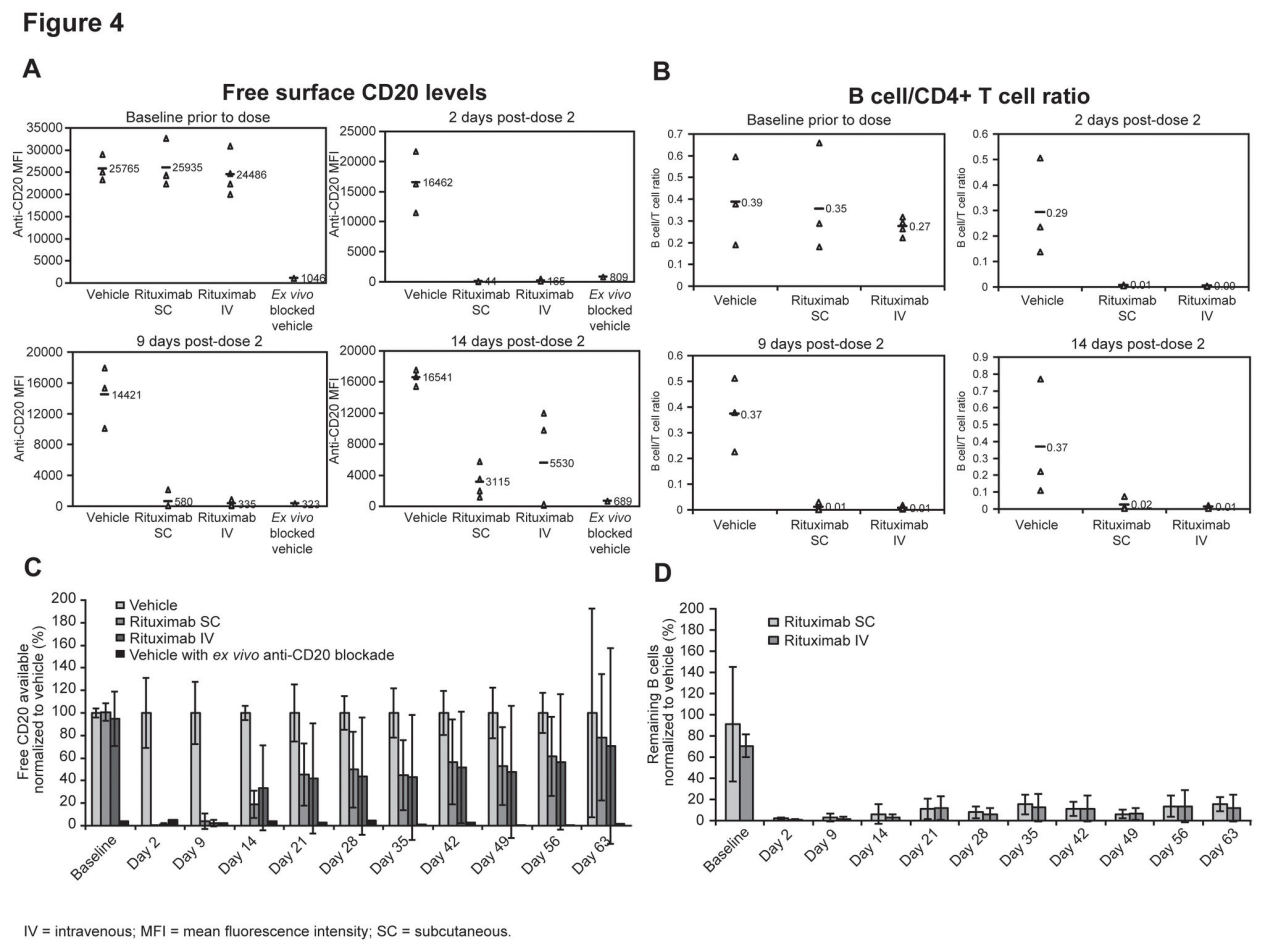
CD20 target coverage and B-cell depletion in Peripheral Blood. PBMC analysis of (**A**) CD20 target coverage as determined by flow cytometric staining for free surface CD20 levels on (B cells identified as in Figure 2A) and (**B**) depletion of those PBMC B-cells at baseline and 2, 9, and 14 days after second dose of subcutaneous or intravenous rituximab as determined by a ratio to CD4+/CD3+ T cells (Individuals animal samples with mean shown (n=4 rituximab treated groups, n=3 PBS vehicle treated groups)); and long-term PBMC analysis of (**C**) percent free surface CD20 target coverage and (**D**) percent remaining B-cells out to 63 days; normalized to PBS vehicle (group mean ± SD (n=4 rituximab treated groups, n=3 PBS vehicle treated groups)) with ex-vivo rituximab treatment of PBS vehicle sample to show maximal target coverage.

Similar to the case with lymphoid tissues, equivalent PBMC B-cell CD20 target coverage and depletion efficacy was observed 2, 9, and 14 days after the second dose of rituximab SC and IV (Figure 4A, B). By day 2, there was >99% reduction in CD20+ B cells compared with baseline for both rituximab SC and IV dosing; by day 9 the reduction for SC versus IV was 98% versus 99%, respectively, and by day 14 it was 88% versus 77%, respectively (Figure 4A). At all time points, B-cell depletion was >94% for both SC and IV dosing (Figure 4B). Furthermore, long-term (2-month) recovery of free CD20 levels was similar, and the duration of B-cell depletion was equally sustained 2 months after rituximab SC and IV dosing (Figure 4C, D). 

### PBMC B-cell subset analysis

**Figure 5:**
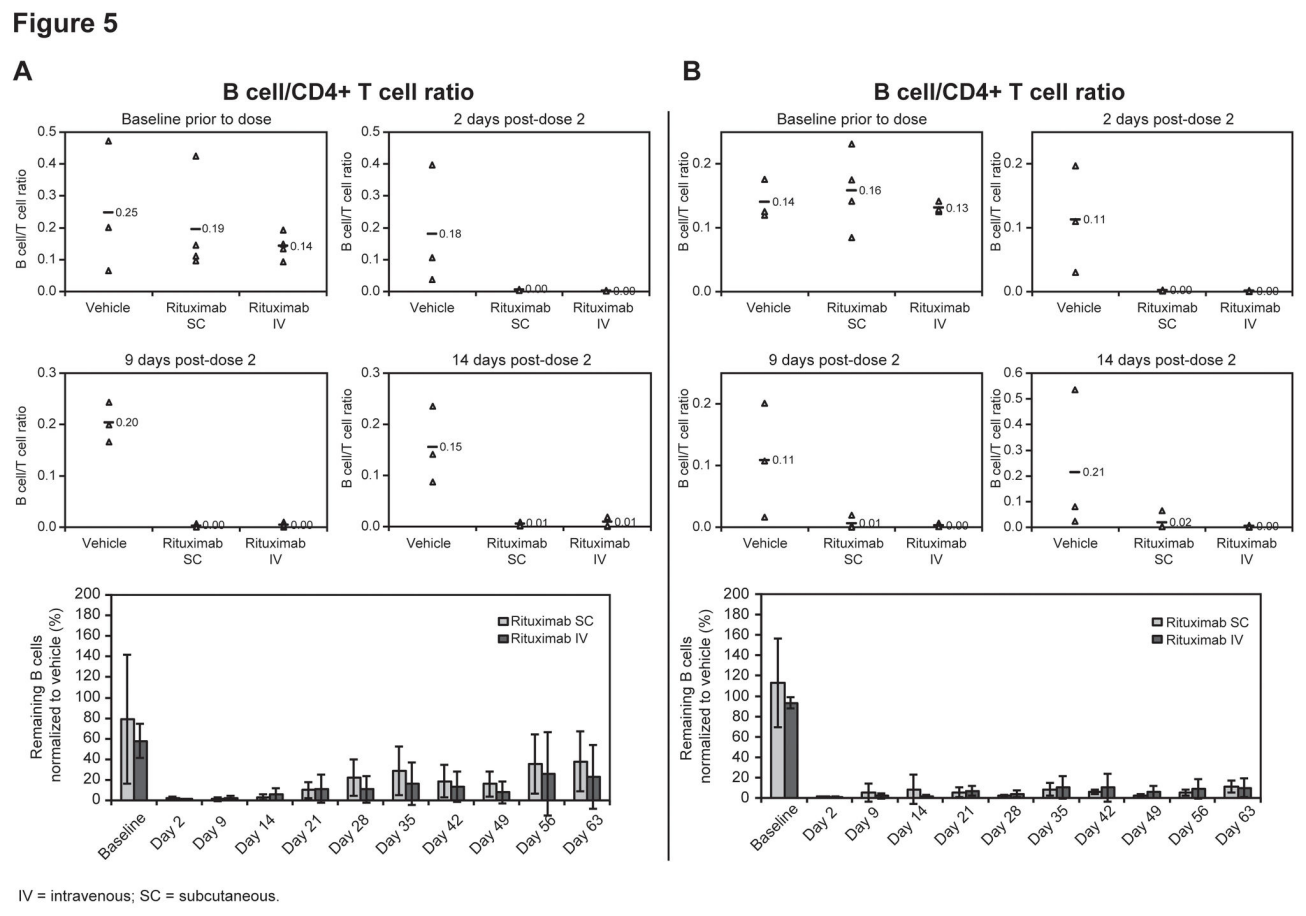
Depletion of Peripheral Blood CD21+ and CD21- B-cell subsets. Short and long-term analysis of PBMC B-cell depletion of (**A**) CD21+ and (**B**) CD21- B-cell subsets (as identified in Figure 2C). Individuals animal samples with mean shown for baseline, day 2, day 9 and day 14 post second dose (top) and percent remaining as normalized to PBS vehicle treated group (bottom) (group mean ± SD (n=4 rituximab treated groups, n=3 PBS vehicle treated groups).

CD21+ and CD21- B-cell subsets were identified within the CD3-/CD4-/CD16-/CD40+ gated B cells from peripheral blood. The PBMC CD21+ B-cell subsets were CD40high and CD20low, and the CD21- cells were CD40low and CD20high, as expected (Figure 2C). Rituximab SC and IV formulations had very similar PBMC B-cell depletion efficacy in both the CD21- and CD21+ B-cell subsets 2, 9, and 14 days after the second dose (Figure 5). At day 2, B-cell depletion was >99% for both SC and IV dosing for both B-cell subsets, falling to 96–100% by day 9 and 89–98% by day 14. In addition, the level of long-term (2-month) B-cell depletion was equally sustained after rituximab SC and IV dosing in both B-cell subsets (Figure 5).

## Discussion

The SC route of administration for rituximab has the potential to decrease administrative healthcare costs while increasing patient safety and convenience. We sought to determine the feasibility of achieving equivalent efficacy (endogenous B-cell depletion) and long-term durability of CD20 target coverage for subcutaneously administered rituximab in comparison with traditional IV dosing in cynomolgus monkeys. Initial PK analysis demonstrated effective dispersion and absorption of rituximab into the blood when a single 20 mg/kg dose of rituximab was administered subcutaneously with a novel formulation containing rHuPH20. The comparative preclinical study then contrasted serum PK titer levels, CD20 target coverage as a PD metric, and B-cell depletion efficacy for SC and traditional IV infusion administration of rituximab given as two 10 mg/kg doses, 7 days apart, approximately equivalent to the standard human dose. We found equivalent target coverage of available surface CD20, and similar B-cell depletion efficacy in both PBMCs and distal secondary lymphoid tissue after SC and IV dosing, indicating that the two routes of administration are equally effective in terms of both target access and efficacy. It is important to note that prior toxicological assessment of single agent rHuPH20 administration in murine and primate studies [12], as well as prior human studies using rHuPH20 in formulation with therapeutic agents such as tocilizumab and analog-insulin [18,19] have not produced any rHuPH20 attributable B cell specific depletion effects that could in effect, mask potential differences in the efficacy of B cell depletion by intravenous versus subcutaneous administered rituximab.

While SC and IV displayed equivalent PD target coverage and depletion efficacy, the one contrasting difference between SC and IV rituximab in these studies was the difference in peak serum C_max_ levels achieved through the two administration routes. These differences were anticipated, as IV administration immediately distributes the bulk dose into the blood circulation, while dissemination from the site of SC administration into the circulation was expected to require time. By 48 hours, SC rituximab reached equal levels to IV (trough), with similar long-term durability and linear PK being found in circulation regardless of the route of administration. With most drugs, peak serum concentrations are often viewed as positive indicators for achieving effective concentrations of drug exposure; however, maximizing time over a dose-efficacy threshold without reaching a dose-limiting toxicity is the optimal goal for therapeutic administration. With antibody-based therapeutics, a further increase in dosage to increase serum concentrations over a dose-efficacy saturation point does not add value, as target coverage is already fully saturated. Moreover, dosing in excess of available effector functions such as phagocytic cells and complement availability may be detrimental to therapeutic benefit if the available antibody bound target is either shaved or endocytosed on target cells prior to the renewed availability of those saturated effector mechanisms. Without surface CD20 bound target antigen/antibody complexes, there would be a continued survival of those antigen downregulated target cells [20]. Previous PK/PD studies for the currently approved IV dose of rituximab indicate that target sites are fully saturated (unpublished observations), and it is believed that maximal clinical benefit is achieved at concentration levels that attain target saturation [21]. As such, half-maximal occupancy of CD20 by ofatumumab (another anti-CD20 targeted antibody) has been shown to attain optimal ADCC activity, but the full CD20 binding occupancy by the agent is required for optimal CDC activity [22)]. This preclinical cynomolgus study supports the hypothesis that SC and IV will result in comparable depletion efficacy, as SC administration attained equivalent PD target saturation and endogenous B-cell depletion to that observed with IV administration in this model species. However, these studies were performed in healthy animals without excess tumor burden, as such there may be differential expectation in the presence of increase antigen availability on B cell tumors that could generate differential concentration dependent tumor penetration and differential antibody clearance prior to available effector functions [20,23,24]. As such, this studyhighlights the importance of measuring the PD of rituximab coverage of surface CD20 as a means to evaluate the achievement of the dose-efficacy saturation point, and suggests that peak serum C_max_ levels are less informative.

PK characteristics of antibody-based biologics are engendered by properties of both ends of the molecule, the epitope-binding antigen-specific end and effector-side mediated Fc receptor (FcR) binding end on the other end, with the former modulating the functional efficiency of the latter. Non-specific linear clearance of antigen-free antibodies is primarily mediated by reticuloendothelial system FcR clearance with a prolonged circulatory retention and half-life mediated by the opposing force of neonatal Fc receptors (FcRn) [25] which retard clearance by binding the effector end and slowly re-releasing functional antibodies back into circulation [26], resulting in larger AUC for free serum antibody exposures [27]. Antigen-specific clearance of antibodies targeting membrane antigens is mediated by both target-cell intrinsic clearance mechanisms and effector cell-dependent mechanisms. Target-cell intrinsic clearance is primarily mediated by endocytotic uptake of antigen/antibody complexes into the cells after surface-target binding, while surface retention post-binding results in the opsonization of target/antibody complexes for FcR-bearing effector cell-dependent phagocytic clearance of the antibody-bound antigens and cells [28]. As therapeutic antibodies possess high binding affinities and low off-rates, they achieve maximal effective exposures upon target saturation. The targeting of soluble antigens that undergo high *de novo* production such as cytokines, and that of antigens that undergo strong endocytic recycling and/or antigenic shedding followed by *de novo* expression of new target antigens, results in an increased demand for available antibody in order to maintain target saturation. However, the targeting of a non-shedding antigen such as CD20 with rituximab results in CD20 antigen internalization and degradation with delayed availability of new CD20 target antigens [29]. This decrease in the availability of new target-antigens. may both limit the further clearance of the therapeutic antibody by antigen mediated mechanisms, but as mentioned previously, may limit the efficacy of the therapeutic if the endocytosis of target antigen/therapeutic complexes occurs during a time of limited effector function availability [20,23,24]. 

A recent two-stage phase Ib study investigating the PK, safety, and tolerability of a single dose of rituximab SC maintenance therapy in 124 patients with follicular lymphoma found that maximum serum concentrations with rituximab SC were achieved between days 2 and 8 [30]. Furthermore, PK parameters were linear with respect to dose over the range of doses investigated (375, 625, and 800 mg/m^2^). Rituximab serum exposure (AUC_0–57_), as well as rituximab serum concentrations on day 28, were comparable between patients administered rituximab SC 625 mg/m^2^ and those given a standard rituximab IV dose of 375 mg/m^2^. While it may be possible to achieve equivalence to IV AUC by elevating SC dosing without reaching a dose-limiting toxicity, we predict from this cynomolgus study that the target-saturating maximal efficacious dose may be lower and achievable by maintaining trough concentration non-inferiority. It would thus be useful to confirm the PD of target coverage across a dose range and time course in future studies so as to identify the minimal dose and frequency required for optimal target coverage.

Rituximab is currently used to treat NHL and CLL using different induction and maintenance treatments that are given by IV infusion over several hours. This prolonged administration time and the adverse events (AEs) related to IV infusion can result in inconvenience for patients, as well as increased demands on healthcare providers and resources. Follicular lymphoma patients who receive rituximab maintenance treatment are particularly affected, as they require infusions every 2 months (starting 2 months after the last dose of induction therapy) for a maximum of 2 years [21]. In the rituximab SC phase Ib trial, doses up to 800 mg/m^2^ were generally well tolerated, and the most commonly reported AEs were administration-associated reactions, including rash, erythema, and mild discomfort [30]. These AEs were reversible and well tolerated, with only one event requiring further treatment, while no symptoms of severe infusion-related reactions associated with cardiac risk or cytokine storms were reported. The authors concluded that overall the AE profile was not notably inferior to what is expected in patients treated with the rituximab IV formulation. It should be noted that the SC administration of biologics in other applications may also be advantageous when the therapeutic index of a molecule is narrow in part due to AEs driven by high initial C_max_ seen during traditional IV administration.

These results demonstrate that despite initial peak serum drug level differences, rituximab SC has comparable durability, pharmacodynamics, and efficacy to rituximab IV. The limited preclinical and clinical data reported to date suggest that rituximab SC administration could potentially simplify treatment for patients by reducing administration times to less than 10 minutes and therefore reduce clinic visit hours. For example, in the phase Ib study a total volume of 4.4–15.0 ml of rituximab SC was administered within a few minutes (average 1.9 ml/min) [30], compared with 4–6 hours for rituximab IV using the standard schedule [3], [17]. Additionally, the SC route of administration may enable treatment to be given in less expensive and more convenient environments for patients. Clinical trials designed to confirm the PK, efficacy, and safety of SC rituximab in patients with follicular NHL and CLL are currently ongoing (clinicaltrials.gov identifiers: NCT01292603, NCT01200758, and NCT00930514) [31-33].
